# Socioeconomic and lifestyle determinants of the prevalence of hypertension among elderly individuals in rural southwest China: a structural equation modelling approach

**DOI:** 10.1186/s12872-021-01885-y

**Published:** 2021-02-02

**Authors:** Li Xiao, Cai Le, Gui-Yi Wang, Lu-Ming Fan, Wen-Long Cui, Ying-Nan Liu, Jing-Rong Shen, Allison Rabkin Golden

**Affiliations:** 1 School of Public Health, Kunming Medical University, 1168 Yu Hua Street, Chun Rong Road, Cheng Gong New City, Kunming, 650500 China; 2 First Affiliated Hospital of Dali Medical University, Dali, China

**Keywords:** Hypertension, Older adults, Socioeconomic status, Lifestyle, China

## Abstract

**Background:**

This study examines the association between socioeconomic and lifestyle factors and the prevalence of hypertension among elderly individuals in rural Southwest China.

**Methods:**

A cross-sectional survey of 4833 consenting adults aged ≥ 60 years in rural regions of Yunnan Province, China, was conducted in 2017. Data on individual socioeconomic status, sleep quality, physical activity level, and family history of hypertension were collected with a standardized questionnaire. Blood pressure, fasting blood glucose, height, weight, and waist circumference were also measured. An individual socioeconomic position (SEP) index was constructed using principal component analysis. Structural equation modelling (SEM) was applied to analyse the association between socioeconomic and lifestyle factors and the prevalence of hypertension.

**Results:**

The overall prevalence of hypertension was 50.6% in the study population. Body fat distribution, including measures of obesity and central obesity, had the greatest total effect on hypertension (0.21), followed by family history of hypertension (0.14), biological sex (0.08), sleep quality (− 0.07), SEP (− 0.06), physical inactivity (0.06), and diabetes (0.06). Body fat distribution, SEP, and family history of hypertension had both direct and indirect effects on hypertension, whereas physical inactivity, diabetes, and sleep quality were directly associated with the prevalence of hypertension. Biological sex was indirectly associated with the prevalence of hypertension.

**Conclusions:**

SEP, body fat distribution, physical inactivity, diabetes, and sleep quality critically influence the prevalence of hypertension. Future interventions to prevent and control hypertension should give increased attention to individuals with low SEP and should focus on controlling diabetes and obesity, increasing physical activity levels, and improving quality of sleep among older adults aged ≥ 60 years in rural Southwest China.

## Background

Hypertension, defined as systolic blood pressure (SBP) ≥ 140 mmHg and/or diastolic blood pressure (DBP) ≥ 90 mmHg or the use of anti-hypertension medication, is an important global public health challenge. Worldwide, the prevalence of hypertension was 26.4% in 2000 and is predicted to increase to 29.2% by 2025 [1]. High SBP accounted for 10.4 million deaths and 218 million disability-adjusted life-years (DALYs) globally in 2017 [2]. The prevalence of hypertension is higher in low- and middle-income countries than in high-income countries, especially among older adults aged ≥ 60 years [3], and hypertension is a preventable risk factor for stroke, coronary heart disease, heart failure, and kidney disease in older adults. The prevalence of hypertension is sharply increasing due to the ageing of the global population, low physical activity levels, and increased BMI [4].

China is the largest developing country in the world, with 1.39 billion people. The population is also ageing, with those aged ≥ 60 years making up 15.5% of the population in 2014 [5], but individuals in this age group are projected to constitute 29.7% of the total population by 2050 [6]. The overall prevalence of hypertension in China increased from 18% in 2002 to 29.6% in 2010 among adults [7]. Furthermore, the prevalence of hypertension increased from 60.1% to 65.2% between 2001 and 2010 among urban residents aged ≥ 60 years [8]. As the population ages and the prevalence of hypertension increases, China is facing an increasingly serious challenge to manage the economic cost of hypertension and its complications.

Yunnan Province, an economically disadvantaged region in southwestern China, is home to 47.4 million people, including 6.1 million adults aged ≥ 60 years. Hypertension has become one of Yunnan’s greatest public health challenges, imposing a considerable economic burden over the past several decades [9]. Large urban–rural gaps in hypertension prevalence exist and deserve more attention from researchers [10].

It is well known that individual educational level, household income, socioeconomic position (SEP), obesity, central obesity, family history of hypertension, diabetes, and physical inactivity have strong associations with the development of hypertension [10]. However, hypertension is caused by a complex interplay of these various factors simultaneously, some with direct effects and some with indirect effects. Few studies have analysed the indirect effect of all of these factors on hypertension and the interaction between the variables [11, 12]. Epidemiologists are increasingly interested in and able to explore all these factors concurrently as a network of multiple pathways leading to disease [13]. Specifically, structural equation modelling (SEM) offers a tool to measure both direct and indirect effects of observational and latent characteristics of observational variables on the risk of diseases [14]. Previous studies have employed SEM to identify risk factors for prediabetes and prehypertension [13, 15]. However, the literature focusing on SEP and lifestyle factors associated with hypertension in rural older adults aged ≥ 60 years in Yunnan is sparse, with prior epidemiological studies focusing on urban areas and overlooking rural communities. We predict socioeconomic factors and lifestyle factors have both direct and indirect effects on hypertension, and the present study aims to fill this knowledge gap.

Specifically, the aim of the present study was to examine the socioeconomic factors (biological sex, age, ethnicity, and SEP) and lifestyle factors (sleep quality, physical inactivity, obesity, central obesity, diabetes, and family history of hypertension) related to the prevalence of hypertension among adults aged ≥ 60 years in rural Yunnan Province using an SEM approach.

## Methods

### Study area and population

We conducted a community-based cross-sectional survey in Yunnan Province. According to the prevalence rate of hypertension in previous studies, we used a formula to calculate the sample size. To ensure a representative study sample, we used a multistage stratified random sampling method. First, all 129 Yunnan counties were classified into three categories, advantaged economic status, normal economic status, and economically disadvantaged, according to per capita gross domestic product (GDP) based on the 2016 Yunnan Statistical Yearbook [16]. One county was then randomly selected from each category. Second, townships in each selected county were divided into two categories according to GDP, and one township was selected from each category for a total of six townships. Third, three villages from each township were selected using the probability proportional to size (PPS) method for a total of 18 villages. Finally, individuals aged ≥ 60 years were selected from each village using a simple random sampling method with random number tables. Older adults with various mental diseases, malignant tumours, acute or chronic infectious diseases, physical limitations or advanced arthritis/arthrosis were excluded.

### Data collection and measurement

Sixteen medical students from Kunming Medical University were selected as interviewers for data collection. All students participated in a training workshop before the commencement of the study to learn how to administer the questionnaire as well as how to measure height, weight, waist circumference, blood pressure (BP), and fasting blood glucose (FBG).

Each study participant who gave informed consent was interviewed by one of these trained interviewers using a pretested and structured questionnaire to collect information on demographic characteristics, socioeconomic status, sleep quality, physical activity, and family history of hypertension.

All methods were performed in accordance with the relevant guidelines and regulations. BP, FBG, height, weight, and waist circumference were measured according to standard protocols. Following the American Heart Association recommendations, BP was measured using a mercury sphygmomanometer on the right arm supported at the level of the heart with the participants in a sitting position after five minutes of rest [17]. Three BP measurements were taken at 5-min intervals. The final recorded measurement was the average of these three BP readings.

FBG was measured using the ACCU-CHEK Perform Glucometer (Roche Diagnostics, Germany) by extracting a small drop of peripheral blood after an overnight fast of at least 10 h.

Height was measured in centimetres to the nearest 0.1 cm using a standard height-measuring ruler. Weight was measured in kilograms to the nearest 0.1 kg with a digital scale (Xiangshan, China). The participants were asked to wear light clothes and stand in an appropriate position with their feet 30 cm apart and their arms hanging at the sides of their body. Waist circumference was measured in centimetres to the nearest 0.1 cm using a measuring tape at the level of midpoint between the lower edge of the 12th costal arch and the anterior superior iliac crest (Additional file 1).

### Definitions

Hypertension was defined as SBP ≥ 140 mmHg or DBP ≥ 90 mmHg, use of anti-hypertension medication during the two weeks prior to the study, and/or self-report of a diagnosis of hypertension by a healthcare professional.

Diabetes was defined as FBG ≥ 7.0 mmol/l, self-reported diagnosis of diabetes by a healthcare professional, or self-reported use of anti-diabetes medication during the previous two weeks.

Body mass index (BMI) was calculated as weight (kg) divided by height squared (m^2^). Obesity was defined as BMI ≥ 28 kg/m^2^ for both men and women. Central obesity was defined as a waist circumference of ≥ 90 cm for males and ≥ 80 cm for females, following World Health Organization (WHO) recommendations for Asian adults [18].

Illiterate was defined as the inability to read or write an understandable full sentence.

Sleep quality was assessed by the Pittsburgh Sleep Quality Index (PSQI). The PSQI consists of 19 self-evaluation questions to assess the quality of sleep of participants in the last month. Seven factors were abstracted, including subjective sleep quality, sleep latency, sleep duration, habitual sleep efficiency, sleep disturbances, use of sleep medications, and daytime dysfunction [19]. Each factor was scored on a 0–3 point scale for a total score of 0–21. High scores refer to worse sleep quality, with poor sleep quality defined as a score of 6 or greater.

Physical inactivity was measured based on sitting time in daily life along with activity intensity during household work according to the health-related behaviours of rural older adults [20]. Specifically, physical inactivity referred to sitting time ≥ 4 h in a day and light activity as a result of household work, whereas physical activity referred to sitting time < 4 h in a day and engagement in moderate or vigorous activity during household work. Sitting time and activity intensity were used as continuous variables and were scored on a 1–3 point scale for a total score of 2–5. High scores refer to physical inactivity, which is defined as a score of 3 or greater, whereas low scores refer to physical activity, which is defined as a score of 2 or less.

Household income, house-construction materials and toilet construction were used as dichotomous variables and scored on a 1- or 2-point scale for a total score of 3–6. High scores, defined as a score of 5 or greater, refer to good household assets, while low scores, defined as a score of 4 or less, refer to poor household assets. Individuals with poor household assets were defined as those with an annual household income in the previous year of less than US$945 and a house made from adobe or stone and without a toilet. Individuals with good household assets were defined as those with an annual household income of more than US$945 and a house made from brick or concrete with a toilet. Good access to medical services was defined as living within a 30-min walk to the nearest medical facility, while poor access to medical services was defined as living more than a 30 min-walk to the nearest medical facility.

A positive family history of hypertension was defined as the presence of hypertension in at least 1 grandparent, parent, or sibling [21].

### Statistical analysis

A chi-squared test was used to compare categorical variables of socioeconomic factors (biological sex, age, ethnicity, education level, household assets and access to medical services) and lifestyle factors (sleep quality, physical inactivity, obesity, central obesity, diabetes, and family history of hypertension) in hypertensive participants. Principal component analysis (PCA) was conducted to derive SEP based on education level, household assets and access to medical services. SEM analysis was used to analyse the relationship between SEP, lifestyle factors, and the prevalence of hypertension. SPSS 22.0 was used for the descriptive analysis and chi-square test, whereas SEM analyses were conducted with AMOS 22.0 and fitted by the maximum likelihood estimation method. P-values of < 0.05 were considered statistically significant.

**Fig. 1:**
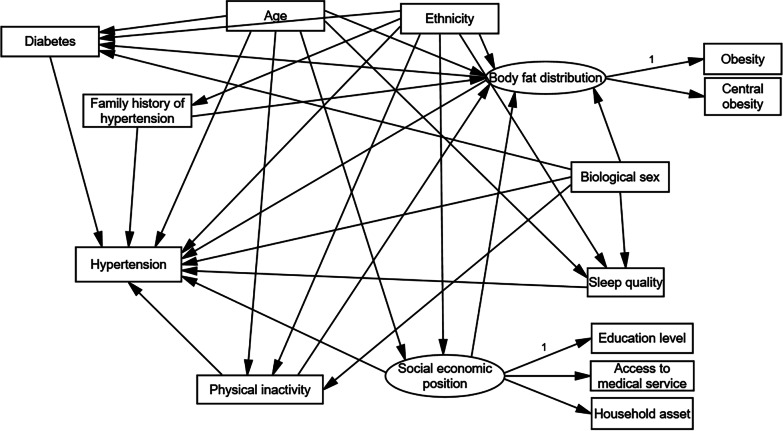
Basic structural equation modeling model of our study

In this study, SEM analyses were conducted in two stages. First, we constructed a hypothesized model based on a literature review [22]. We constructed two latent variables, including SEP and body fat distribution. SEP incorporated educational level, household assets, and access to medical services, whereas body fat distribution was composed of obesity and central obesity. The basic model is shown in Fig. 1. The observed variables are represented by rectangles, whereas latent variables are represented by ellipses. Direct effects are depicted as a line with an arrow from a variable to other variables. In this model, socioeconomic characteristics—including SEP (educational level, household assets and access to medical services), age (in years), biological sex (female or male), ethnicity (Han majority and ethnic minority), and family history of hypertension (yes or no)—were considered exogenous variables. Lifestyle factors—including body fat distribution (obesity and central obesity), physical inactivity (sitting time in daily life and activity intensity during household work), sleep quality (good or poor), and diabetes (yes or no)—were considered endogenous variables that can be affected by both exogenous and other endogenous variables. Hypertension was measured as dichotomous variable (yes = 1, no = 0) in this model. We hypothesized 28 paths that directly or indirectly affected hypertension. These paths originated from 7 observed variables and 2 latent variables. Biological sex, age, ethnicity and family history of hypertension are determined at birth but still impact the prevalence of hypertension directly or indirectly through other variables. Second, we tested the significance of all exogenous variables and endogenous variables on hypertension and analysed the direct and indirect effects of all variables on hypertension. The aim of this hypothesized model was to identify whether SEP and modifiable factors, such as body fat distribution, physical inactivity, sleep quality and diabetes, affect hypertension.

Modification indices were used to evaluate and select appropriate paths for the best fitted model. We calculated goodness-of-fit indices, including root mean square error of approximation (RMSEA), goodness-of-fit index (GFI), comparative fit index (CFI), Tacker-Lewis index (TLI), and weighted root mean square residual (WRMR), to evaluate the best fit model. Direct, indirect, and total effects were calculated and recorded.

## Results

A total of 5000 individuals aged ≥ 60 years were involved in the survey. Of these, after excluding 171 individuals with missing variables, 4833 participants were included in the final analysis. The response rate was 96.6%. The general characteristics of the target participants are shown in Table 1. The older adults aged ≥ 60 years who were included in the survey included 2198 (45.5%) males and 2635 (54.5%) females. Han majority and ethnic minority participants constituted 83.3% and 16.7% of the total population, respectively, whereas older adults aged ≥ 60 years with poor household assets and poor access to medical services accounted for 28.6% and 21.4% of the participants, respectively. A total of 65.0% of older adults had a higher educational level. The proportions of older adults suffering from physical inactivity, poor sleep quality, obesity, central obesity, diabetes, and family history of hypertension were 51.1%, 46.7%, 6.6%, 52.8%, 10.2% and 15.3%, respectively.

**Table 1:** Characteristics of study participants

Variables	n	%	95% CI
Biological sex			
Male	2198	45.5	(44.0, 46.9)
Female	2635	54.5	(53.1, 56.0)
Age group			
60–64 years	1326	27.4	(26.1, 28.7)
65–69 years	1284	26.6	(25.4, 27.9)
70–74 years	1047	21.7	(20.4, 22.8)
≥ 75 years	1176	24.3	(23.2, 25.6)
Ethnicity			
Han	4026	83.3	(82.3, 84.3)
Minority	807	16.7	(15.7, 17.7)
Educational level			
Illiterate	1690	35.0	(33.7, 36.4)
Primary (grade 1–6) or higher	3143	65.0	(63.6, 66.3)
Household assets			
Good	3453	71.4	(70.1, 72.7)
Poor	1380	28.6	(27.3, 29.9)
Access to medical service			
Good	3797	78.6	(77.3, 79.7)
Poor	1036	21.4	(20.3, 22.7)
Sleep quality			
Good	2574	53.3	(51.7, 54.6)
Poor	2259	46.7	(45.4, 48.3)
Physical inactivity			
Yes	2472	51.1	(49.8, 52.6)
No	2361	48.9	(47.4, 50.2)
Obesity			
Yes	321	6.6	(5.9, 7.4)
No	4512	93.4	(92.6, 94.1)
Central obesity			
Yes	2551	52.8	(51.3, 54.2)
No	2282	47.2	(45.8, 48.7)
Family history of hypertension			
Yes	738	15.3	(14.2, 16.3)
No	4095	84.7	(83.7, 85.8)
Diabetes			
Yes	494	10.2	(9.3, 11.2)
No	4339	89.8	(88.8, 90.7)
Total	4833	100.0	

Table 2 presents the prevalence of hypertension among rural adults in Yunnan Province aged ≥ 60 years according to socioeconomic and lifestyle factors. The overall prevalence of hypertension was 50.6%. Older adults aged ≥ 60 years with poor household assets, poor access to medical services, poor sleep quality, physical inactivity, and family history of hypertension had a higher prevalence of hypertension than their counterparts; additionally, older adults aged ≥ 60 years with obesity, including central obesity, and diabetic older adults had a higher prevalence of hypertension than their counterparts (*P* < 0.01).

**Table 2:** Prevalence of hypertension among rural adults aged ≥ 60 years in Yunnan Province, China, according to socioeconomic and lifestyle factors

Variables	n	%	95% CI	*P* value
Biological sex				0.001
Male	1047	47.6	(45.5, 49.7)	
Female	1400	53.1	(51.2, 54.9)	
Age group				0.001
60–64 years	591	44.6	(42.0, 47.3)	
65–69 years	649	50.5	(47.8, 53.3)	
70–74 years	562	53.7	(50.9, 56.7)	
≥ 75 years	645	54.8	(52.2, 57.6)	
Ethnicity				0.015
Han	2007	49.9	(48.4, 51.3)	
Minority	440	54.5	(51.1, 57.8)	
Educational level				0.220
Illiterate	876	51.8	(49.4, 54.3)	
Primary (grade 1–6) or higher	1571	50.0	(48.3, 51.8)	
Household assets				0.004
Good	1703	49.3	(47.6, 50.9)	
Poor	744	53.9	(51.2, 56.6)	
Access to medical service				0.013
Good	1887	49.7	(48.1, 51.2)	
Poor	560	54.1	(50.9, 57.1)	
Sleep quality				0.001
Good	1191	46.3	(44.3, 48.2)	
Poor	1256	55.6	(53.5, 57.6)	
Physical inactivity				0.001
Yes	1349	54.6	(52.6, 56.6)	
No	1098	46.5	(44.5, 48.6)	
Obesity				0.001
Yes	224	69.8	(64.8, 74.6)	
No	2223	49.3	(47.8, 50.8)	
Central obesity				0.001
Yes	1500	58.8	(56.9, 60.7)	
No	947	41.5	(39.5, 43.5)	
Family history of hypertension				0.001
Yes	501	67.9	(64.7, 71.1)	
No	1946	47.5	(46.0, 49.2)	
Diabetes				0.001
Yes	318	64.4	(60.2, 68.6)	
No	2129	49.1	(47.5, 50.4)	
Total	2447	50.6		

The PCA results indicated satisfactory reliability (Kaiser–Meyer–Olkin (KMO) = 0.529), and the Bartlett test of sphericity was statistically significant (*P* < 0.001). The three variances associated with SEP accounted for 39.0% of the total variance. As shown in Table 3, the final model reached the model fit indices, which are generally considered to indicate a reasonable model fit to the data, including RMSEA, GFI, CFI, TLI, and WRMR. We tested 797 models by a machine learning system and identified the final model that fit the hypothesis most appropriately. The following changes were made from the basic model to create the final model. First, we dropped age and ethnicity from the basic model, as the modification indices revealed that the path was not statistically significant (*P* ≥ 0.05). Second, we dropped the direct effect of biological sex on diabetes and hypertension and of physical inactivity on body fat distribution based on the fit of the model according to the goodness-of-fit index.

**Table 3:** Goodness-of-fit index of the structural equation modelling

Goodness-of-fit	Reference value	Value
Root mean square error of approximation (RMSEA)	< 0.05	0.024
Coefficient of determination (GFI)	> 0.90	0.995
Comparative fit index (CFI)	> 0.90	0.945
Tacker-Lewis index (TLI)	> 0.90	0.927
Weighted root mean square residual (WRMR)	< 0.05	0.004

**Fig. 2:**
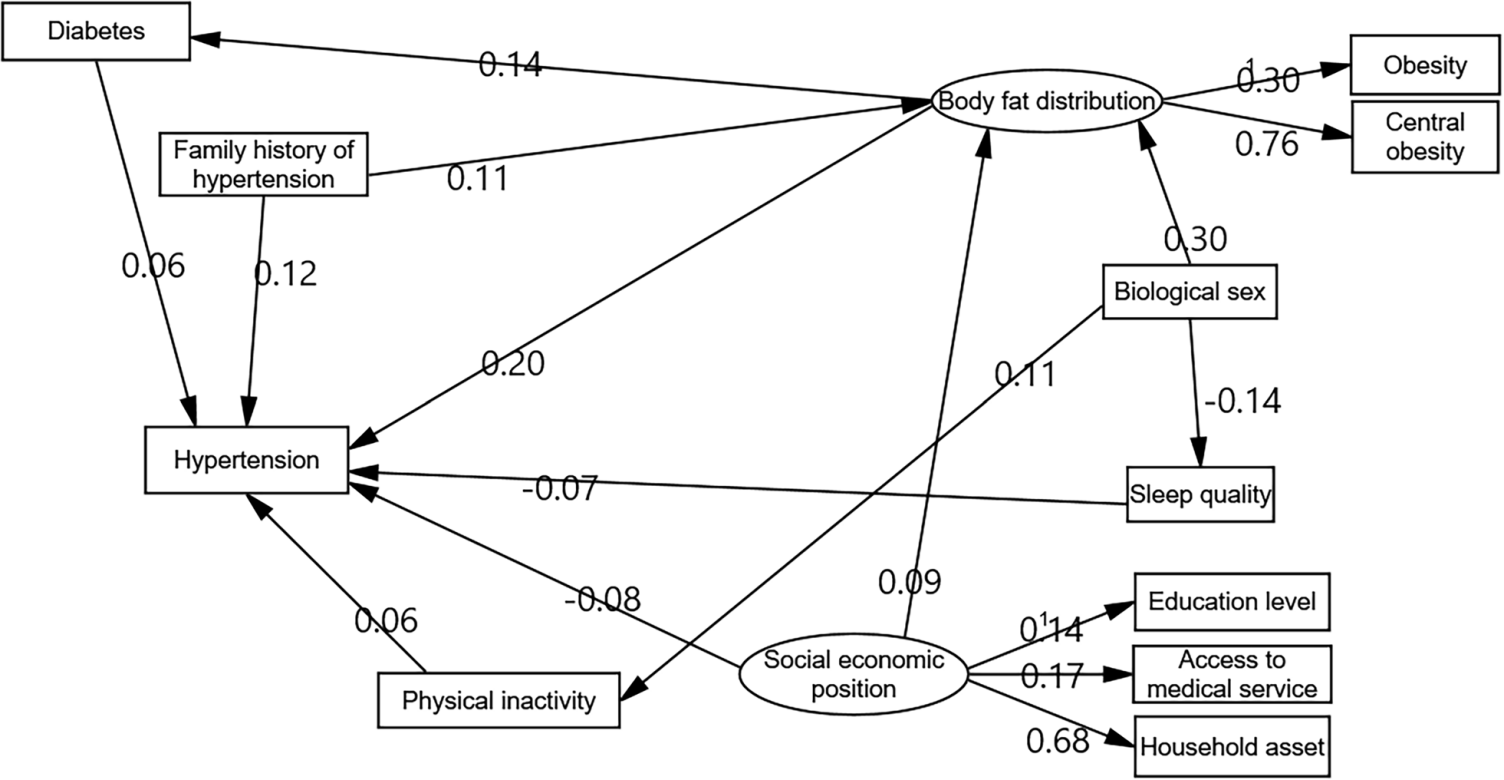
Final structural equation modeling model of associated factors of hypertension among rural adults aged ≥ 60 years in Yunnan Province, China

Figure 2 shows the final SEM model of factors associated with hypertension. Whereas biological sex (female = 1, male = 0) had a negative relationship with sleep quality (− 0.14, *P* < 0.001), it had a positive relationship with body fat distribution (0.30, *P* < 0.001) and physical inactivity (0.11, *P* < 0.001). Higher socioeconomic status had a negative association with hypertension (− 0.08, *P* = 0.001) and a positive relationship with body fat distribution (0.09, *P* = 0.008). The effect of body fat distribution on hypertension (0.20, *P* < 0.001) was greater than that of diabetes (0.14, *P* < 0.001). A family history of hypertension had similar effects on hypertension (0.12, *P* < 0.001) and body fat distribution (0.11, *P* < 0.001). Diabetes (0.06, *P* < 0.001) and physical inactivity (0.06, *P* < 0.001) had the same positive relationship with hypertension, while sleep quality (− 0.07, *P* < 0.001) had a negative effect on hypertension.

**Fig. 3:**
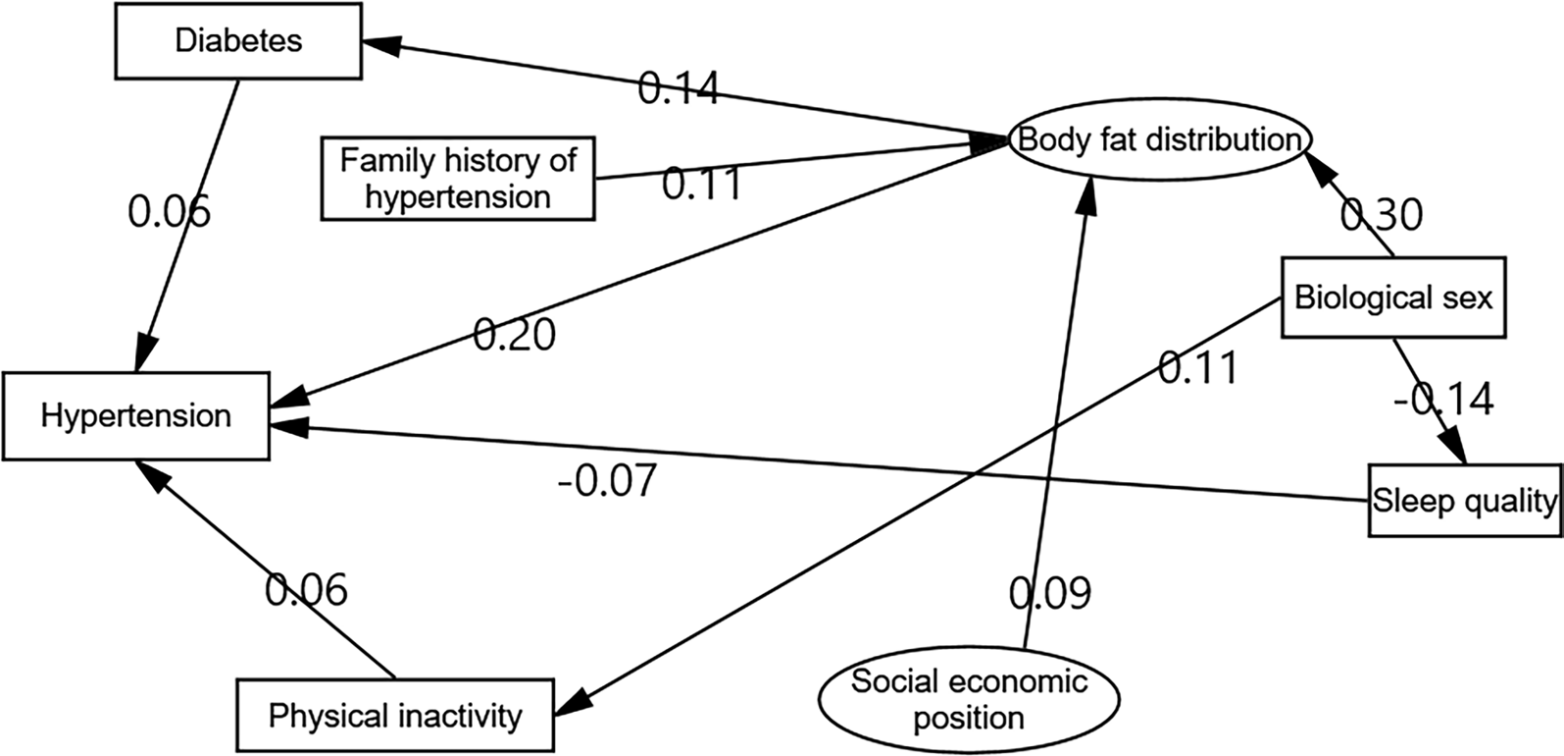
Indirect effects of associated factors of hypertension based on structural equation modeling model among rural adults aged ≥ 60 years in Yunnan Province, China

Table 4 presents the direct, indirect, and total effects of the studied variables on hypertension. Overall, body fat distribution had the greatest total effect on hypertension (0.21), followed by family history of hypertension (0.14), biological sex (0.08), sleep quality (− 0.07), SEP (− 0.06), physical inactivity (0.06), and diabetes (0.06). Figure 3 shows that biological sex alone had an indirect effect on hypertension through mediators, including physical inactivity, sleep quality, and body fat distribution.

**Table 4:** Direct, indirect, and total effects of variables on hypertension

Variables	Direct	Indirect	Total
Family history of hypertension	0.12	0.02	0.14
Physical inactivity	0.06	No path	0.06
Diabetes	0.06	No path	0.06
SEP	− 0.08	0.02	− 0.06
Biological sex	No path	0.08	0.08
Sleep quality	− 0.07	No path	− 0.07
Body fat distribution	0.20	0.01	0.21

## Discussion

To our knowledge, this is the first study to use SEM to examine the direct and indirect effects of socioeconomic and lifestyle determinants on hypertension in China. The findings indicate that body fat distribution, SEP, and family history of hypertension have both direct and indirect effects on hypertension, while physical inactivity, diabetes, and sleep quality are directly associated with hypertension and biological sex is indirectly associated with hypertension.

Our study indicated noticeably high prevalence rates of physical inactivity and poor sleep quality among older adults aged ≥ 60 years in rural Southwest China. The prevalence of physical inactivity in our participant population was also higher than the 27.5% global prevalence [20]. Furthermore, the prevalence of poor sleep quality was higher than that observed in previous studies in China [23]. These high prevalence rates may result from the demographic transition caused by the economic development currently underway in China as well as lack of awareness of the impact of lifestyle choices on chronic disease among older adults aged ≥ 60 years in rural Yunnan Province. Correspondingly, as these central determinants of blood pressure lead to a higher prevalence of hypertension among the studied population, the prevalence of hypertension was similar to the reported prevalence rate of 44.6–60.1% in the China Hypertension Survey [24] and similar to the worldwide prevalence of 33–59% among individuals aged 40–79 years [25].

SEM was used to construct a complex theoretical model that reflected reality. The study indicated that body fat distribution was the most important risk factor that directly affected the development of hypertension in our study. Furthermore, the high prevalence of obesity and central obesity directly contributed to the occurrence of hypertension, which is consistent with the WHO report [3]. Body fat distribution also indirectly affected hypertension via diabetes. This may be due to alterations at hormonal, inflammatory, and endothelial levels that result from obesity and central obesity [26]. The findings suggested that it is urgently necessary to advise individuals, especially for older adults with diabetes, to engage in weight management programmes and control their waist circumference to prevent hypertension.

The study established that high SEP was a protective factor that directly affected hypertension. This finding is inconsistent with studies conducted in Sudan [27] and South Africa [28]. This may result from the fact that older adults with better SEP had better health awareness and more opportunities to prevent, diagnosis, treat, and manage hypertension in Yunnan Province than in Sudan and South Africa. SEP also negatively affected hypertension through body fat distribution. This result indicated that older adults with better SEP may be more likely to have obesity or central obesity, which impacts the prevalence of hypertension. Thus, the findings indicate that older adults with lower SEP and older adults with higher SEP who are less conscious of body fat control need more effective prevention and intervention programmes for hypertension prevention.

The present study also found that poor sleep quality had a direct effect on the prevalence of hypertension. The contribution of sleep quality to the development of hypertension has been established in previous research [29]. Poor sleep quality is common in older populations, and our results of a high prevalence of poor sleep quality were similar to those of a cross-sectional survey conducted in Iran [30]. Given this finding, improving sleep quality among Yunnan residents could have a positive effect on the prevention of hypertension.

Physical inactivity was positively and directly associated with the prevalence of hypertension in our study. This finding aligns with previous studies conducted in China [31], Greece [32], and the UK [33]. Our study suggested that increasing moderate and vigorous physical activity as well as reducing sitting time is important for the prevention of hypertension among rural older adults. Moreover, we found that a family history of hypertension also directly affected the prevalence of hypertension, a result consistent with previous research [34]. This study thus indicates that those with a family history of hypertension should have regular screenings for hypertension and undergo lifestyle interventions for hypertension prevention.

In our study, diabetes had a positive, direct effect on the prevalence of hypertension. In the basic model of our study, diabetes was also found to be affected by biological sex, age, ethnicity, and body fat distribution. Finally, age, biological sex, and ethnicity were eliminated from the model because the paths were not significant. This is consistent with previous research that found that the prevalence of diabetes and hypertension was associated with similar risk factors [35]. This finding also suggests that maintaining proper body fat distribution promotes hypertension and diabetes prevention.

Our study also showed that the effect of biological sex on hypertension was mediated by body fat distribution, physical inactivity, and sleep quality. This result indicated that the factors that made hypertension more frequent in women were related to differences in risk factors and life expectancy between men and women. This was contrary to a previous study conducted in China [36].

### Limitations

The following limitations of the present study should be noted. First, haemoglobin A1C was not measured, and the diagnosis of diabetes was solely based on FBG tests or self-reported. This may underestimate the prevalence of diabetes among the study population. Second, physical exercise was self-reported in the study questionnaire, which could introduce inaccuracies, as it was subject to participant recall. Physical activity should be measured in metabolic equivalents (METs) in the future to strengthen the analysis results. Third, data on participants’ diets, mental factors renal function, uric acid and lipid parameters were not included in the present study, and they are important factors in the development of hypertension. Fourth, we did not consider using multilevel models to handle cluster data or survey weights in the statistical model in our study, and we should do so in future work to further explore the results of our analysis. Fifth, hypertension could directly affect low socioeconomic position, but we could not consider this situation in our study. Finally, the findings were based on a study of three counties, which could limit the ability to generalize the results to the entire province.

## Conclusions

In sum, based on the findings that SEP, body fat distribution, physical inactivity, diabetes, and sleep quality have significant influences on the prevalence of hypertension, future interventions to prevent and control hypertension among rural Chinese older adults should focus on those with low SEP, while interventions to control diabetes should focus on body fat distribution, increasing physical activity levels and improving the quality of sleep.

## Supplementary Information

**Additional file 1:** Survey questionnaire.

## Data Availability

The datasets used and/or analysed during the current study are available from the corresponding author on reasonable request.

## Abbreviations

BP: Blood pressure
BMI: Body mass index
CFI: Comparative fit index
DALYs: Disability-adjusted life-years
FBG: Fasting blood glucose
GDP: Gross domestic product
GFI: Goodness-of-fit index
KMO: Kaiser–Meyer–Olkin
PCA: Principal component analysis
PSQI: Pittsburgh Sleep Quality Index
PPS: Probability proportional to size
RMSEA: Root mean square error of approximation
SEM: Structure equation modelling
SEP: Socioeconomic position
TLI: Tacker-Lewis index
WHO: World Health Organization
WRMR: Weighted root mean square residual
