# Supplementary material for: Socioeconomic and lifestyle determinants of the prevalence of hypertension among elderly individuals in rural southwest China: a structural equation modelling approach
Source: BMC Cardiovasc Disord. 2021 Feb 2;21:64. doi: 10.1186/s12872-021-01885-y (PMC7851929; doi:10.1186/s12872-021-01885-y)
Supplement: Supplementary file 1 — Additional file 1: Survey questionnaire. [file 12872_2021_1885_MOESM1_ESM.docx]

The questionnaire of common chronic diseases among older adults in Yunnan province

Number of questionnaire: County: Township:

Village: Account number:

Investigator: Investigation time:

Guidance: The following is a questionnaire about your health condition. We want to know your health condition through this survey and will provide you a free physical examination. Do you agree?

**Demographic characteristics**

1. Your birthday: _______ month _______day _______ year，or age _______ or Zodiac _______.

ID card Number：_______ ；or health insurance number: _______ ;

Telephone number:_______

1. Gender: male:_______; female:_______ ;
2. How many years do you get education?
3. Ethnicity: ①Han majority; ②minority_______
4. How many people are there in your family?_______ ;What was the total household income last year?______.What is the per capita income last year?______.
5. Do you use running water in your house?______.
6. Do you have a toilet in your house?______.
7. What is your living condition?①brick ②concrete ③adobe ④stone ⑤other_______. how much time do you walk from your home to the nearest medical facility? ①≥30min ②<30min

**Chronic disease condition**

1. Do you diagnose with hypertension? Yes _______;No _______.
2. What is your hypertension diagnosis institution? ①village clinic ② township clinic ③county/municipal hospital ④ provincial hospital ⑤other_______
3. Do you diagnose with complication of hypertension?

①no ②stroke ③hypertension heart disease ④hypertensive nephropathy ⑤miocardial infarction ⑥coronary heart disease ⑦other_______

1. Do you diagnose with diabetes? Yes _______;No _______.

**Family history of chronic diseases**

1. Next, I'm going to ask some questions about your family member’s health. Including living or dead family members, have doctors ever told your immediate family members (parents, brothers, sisters and children) with diabetes?

**①** No ② Yes ③ i don’t known

1. Have doctors ever told your immediate family members (parents, brothers, sisters and children) with hypertension?

**①** No ② Yes ③ i don’t known

**Physical activity**

15. In your work, agricultural and domestic activities, which of the following types of physical activity intensity does your work belong to or approach?

① High intensity labor (such as manual harvesting, digging, plowing etc.)which requires a lot of physical strength, or causes obvious acceleration of breathing and heartbeat

② Moderate intensity labor (such as washing clothes, cleaning, carrying water, etc.) requires moderate physical strength, or causes slight increase in respiration and heartbeat

③ Light intensity labor (75% of working time is sitting or standing, 25% of time is standing to work)

1. Sitting time in work or daily life (8 hours): ① almost all; ② more than 4 hours; ③ less than 4 hours; ④ almost none

**Physical examination**

Note: before the examination, the participates should sit and rest for more than five minutes.

1. height_______cm; 18. weight _______cm;
2. waist circumference_______cm; 20. hip circumference_______cm;
3. blood pressure: systolic blood pressure 1 _______mmHg；

diastolic blood pressure1 _______mmHg；

systolic blood pressure 2 _______mmHg；

diastolic blood pressure2 _______mmHg；

systolic blood pressure 3 _______mmHg；

diastolic blood pressure3 _______mmHg；

1. Do you eat any food today? **①** No ② Yes
2. Are your blood samples collected today?**①** No ② Yes
3. Blood glucose level: ____________mmol/L.

**Pittsburgh sleep quality index (PSQI) scale**

Instructions: the following questions are about your sleep status in the last month. Please select or fill in the answers that best meet your actual situation in the past month, and the parts with horizontal lines need to be filled in. Please answer the following questions:

1.For nearly a month, it is usually at _______o'clock to go to bed at night

2. In the past month, it usually takes _______minutes from bed to sleep (please choose)

① ≤ 15 minutes ②16-30 minutes ③ 31-60 minutes ④ > 60 minutes

3. In the past month, I usually get up at _______o'clock in the morning.

4. In the past one month, i actual sleep _______hours per night (not equal to bed time).

Choose one of the following questions that best suits your situation and tick "√".

5. In the past month, the following conditions affect sleep and worry:

a. Difficulty in falling asleep (refers to not falling asleep within 30 minutes)

①none ② < 1 / week ③1-2 times / week ④≥ 3 times / week

Who choose ②③ ④Answer: do you think the main reasons for your difficulty in falling asleep are:

① No reason ② physical discomfort, please explain ③ environmental factors, please explain ④ psychological factors, please explain⑤others

b. Easy to wake up or wake up early at night ①no ② < 1 / week ③1-2 times / week ④ ≥ 3 times / week

Who choose ②③④answers:

Ba. Do you think that the main reasons that cause you to wake up easily at night are as follows:

① No reason ②physical discomfort, please explain ③environmental factors, please explain ④psychological factors, please explain ⑤ others

Bb. Do you think that the main reasons that cause you to wake up early at night (unable to fall asleep again after waking up in the middle of the night or after waking up in the morning) are:

① No reason②physical discomfort, please explain ③environmental factors, please explain ④ psychological factors, please explain ⑤others

c. Going to the toilet at night ① none ② < 1 / week③ 1-2 times / week ④≥ 3 times / week

d. Dyspnea ① no ② < 1 / week ③1-2 times / week ④≥ 3 times / week

e. High cough or snoring① no② < 1 / week ③1-2 times / week ④ ≥ 3 times / week

f. Feeling cold ① no ② < 1 time / week ③1-2 times / week④ ≥ 3 times / week

g. Feeling heat ① no ②< 1 time / week ③ 1-2 times / week ④ ≥ 3 times / week

h. Nightmares ① no ② < 1 / week ③1-2 times / week ④≥ 3 times / week

i. Pain and discomfort①no ② < 1 time / week ③1-2 times / week ④≥ 3 times / week

j. Other sleep related events ① none ②< 1 times / week ③1-2 times / week ④ ≥ 3 times / week, if any other situation, please explain_______;

6A. In the past month, generally speaking, you think your sleep quality is:

①very good②good ③poor④very poor

6B. The main problems of your poor sleep are: ①difficulty in falling asleep; ②waking up early (unable to fall asleep again after waking up in the middle of the night or in the early morning) ③easy to wake up at night (easy to wake up in the middle of sleep and continue to fall asleep after waking up)

7. Hypnosis with drugs in the past month:① none ② < 1 time / week③1-2 times / week ④ )≥ 3 times / week

Who choose ② ③④ answers:

7a. Source of your hypnotic drugs: ①doctors prescribe drugs; ② purchase them from pharmacies; ③other channels

7b. Do you think hypnotics are helpful for your sleep? ①Yes ② No

7C. Do you have mental / physical withdrawal symptoms when you stop using hypnotics? ①No ② Yes, please explain

8. Do you often feel sleepy in the past month? ①None②< 1 / week ③1-2 times / week ④ ≥ 3 times / week

9. In the past month, have you been short of energy? ① No ② occasionally ③sometimes ④often
